# Neuron‐Derived Extracellular Vesicles: Emerging Biomarkers and Functional Mediators in Alzheimer's Disease, With Comparative Insights Into Neurodevelopment and Aging

**DOI:** 10.1002/dneu.22984

**Published:** 2025-06-12

**Authors:** Ceren Perihan Gonul, Bilge Karacicek, Sermin Genc

**Affiliations:** ^1^ Izmir Biomedicine and Genome Center Izmir Turkey; ^2^ Izmir International Biomedicine and Genome Institute Dokuz Eylul University Izmir Turkey; ^3^ Department of Neuroscience, Institute of Health Sciences Dokuz Eylul University Izmir Turkey

**Keywords:** aging, Alzheimer's disease, biomarker, extracellular vesicle, neurodevelopment, neuron‐derived extracellular vesicles

## Abstract

Alzheimer's disease (AD) is one of the most common neurodegenerative disorders characterized by the accumulation of amyloid‐β (Aβ) peptide and phosphorylated tau protein in the brain. Despite intensive efforts, early diagnosis and monitoring of AD remain challenging due to the lack of reliable biomarkers that can detect the disease in its preclinical stages. As a result, there exists a requirement for novel approaches to the diagnosis and treatment of the disease. Extracellular vesicles provide the transfer of Aβ peptides and tau proteins between the cells and participates in the spreading/propagation of disease pathology. Neuron‐derived extracellular vesicles (NDEVs) that are found in plasma have emerged as promising candidates, especially for biomarker studies on neurodegenerative diseases because they are reachable and comparable with cerebrospinal fluid (CSF) studies. In addition to known proteins, synaptic proteins, transcription factors, or microRNAs have been suggested as new biomarkers, aiming to help differential or early diagnosis. Beyond their involvement in AD pathology, NDEVs also play essential roles in neurodevelopment and aging by mediating cell‐to‐cell communication and regulating processes such as synaptic formation, neuronal differentiation, and neuroinflammation. Age‐related alterations in EV composition and secretion may contribute to the decline in neuroplasticity, thereby increasing susceptibility to neurodegenerative diseases like AD. Several challenges such as heterogeneous isolation of NDEVs limit the widespread clinical application of them as biomarkers for AD. Furthermore, the lack of standardized protocols for vesicle isolation and molecular analysis poses a barrier to reproducibility and clinical validation. The aim of this review is to elucidate the role of NDEVs in AD pathogenesis in comparison with their functions in neurodevelopment and aging, evaluate their potential as biomarkers for early diagnosis, while addressing the challenges in their isolation, characterization, and clinical application.

**Abbreviations**
AD: Alzheimer's disease
APOA2: Apolipoprotein II‐A
APP: amyloid precursor protein
Aβ: amyloid‐β
BBB: blood–brain barrier
C7: Complement component 7
CN: cognitively normal
CNS: central nervous system
CSF: cerebrospinal fluid
DM: diabetes mellitus
ELISA: enzyme‐linked immunosorbent assay
EV: extracellular vesicle
fMRI: functional magnetic resonance imaging
FTD: frontotemporal dementia
GAP43: growth‐associated protein 43
hiPSC: human induced pluripotent stem cell
ISEV: International Society of Extracellular Vesicles
L1CAM: L1 cell adhesion molecule
MCI: mild cognitive impairment
miRNA: microRNA
MMSE: mini‐mental state examination
MRI: magnetic resonance imaging
NCAM: neural cell adhesion molecule
NDEV: neuron‐derived extracellular vesicles
NFT: neurofibrillary tangles
NGS: next‐generation sequencing
NLGN1: Neuroligin 1
NLGN3: Neuroligin 3
NPTX2: Neuronal pentraxin 2
NRGN: neurogranin
NRXN2a: Neurexin 2a
P2RX7: P2X purinoceptor 7
PET: positron emission tomography
p‐tau: phosphorylated tau
TF: transcription factor
TNF‐α: tumor necrosis factor‐alpha
t‐tau: total tau
TXNIP: thioredoxin‐interacting protein
WT: wild‐type
ZYX: zyxin

## 1 Introduction

Alzheimer's disease (AD) is a neurodegenerative disorder that can also be described as a type of dementia. It gradually progresses with age, and the symptoms are generally cognitive decline, loss of memory, distorted language, and loss of judgment (Khan et al. 2020). Neuropathological hallmarks of AD are amyloid plaque generated from the accumulation of amyloid‐β (Aβ) peptides, neurofibrillary tangles (NFTs) caused by tau hyperphosphorylation, and neural loss (Li, Gui et al. 2020). Inflammation caused by microglia plays an important role in AD pathogenesis (Hansen et al. 2018).

For AD‐associated neurocognitive disorders, neurodegenerative changes in the brain start much earlier than the first observation of the symptoms (American Psychiatric Associaction 2013). Therefore, early diagnosis is important. Common approaches for diagnosis of AD include structural imaging of brain atrophy using magnetic resonance imaging (MRI), functional MRI (fMRI), imaging of amyloid or tau through positron emission tomography (PET), and the assessment of cerebrospinal fluid (CSF) or blood biomarkers (Dong et al. 2020; Frisoni et al. 2010). Proteins present in CSF or blood, such as low Aβ_(1–42)_ levels and increased total or phosphorylated tau (p‐tau) levels, are also regularly used as biomarkers for the diagnosis of cognitive impairments linked to AD (Hampel and Blennow 2004). The identification of sensitive biomarkers that reflect the pre‐symptomatic neurodegenerative changes remains essential. Testing different biomarkers from both CSF and blood can yield more accurate results (Alawode et al. 2021). Neuron‐derived extracellular vesicles (NDEVs) present in the bloodstream are notable sources for neurodegeneration biomarkers, as they can transport information from the brain into periphery.

EVs are lipid‐bilayer‐enclosed, nanoscale vesicles that are spontaneously expelled from cells (Li, Gui et al. 2023). They range in diameter from around 30 nm to 10 µm and are generally divided into four sub‐categories: ectosomes (∼100–1000 nm), apoptotic bodies (∼1000–5000 nm), exosomes (∼30–150 nm), and oncosomes (∼1–10 µm) (Kong et al. 2023; Wang et al. 2023). Nonetheless, current technologies struggle to differentiate between the two subtypes because of their partial overlap, and the precise markers identifying each EV subtype remain a matter of debate. In order to prevent misunderstandings or inaccurate definitions, the general word “EVs” is used in this study to describe exosomes, multivesicular bodies, and other terms in accordance with the most recent recommendations of the International Society of Extracellular Vesicles (ISEV) (Li, Gui et al. 2023).

EVs can encapsulate and convey the proteins and nucleic acids of the host cell at the time of release. Upon uptake by recipient cells, these vesicles can modify cellular functions, thereby initiating a chain of events that disrupts homeostasis. EVs are found in many body fluids, such as blood, CSF, saliva, and urine. Analyzing specific cargos may be used to explore this particular biological niche, offering a “liquid biopsy snapshot” of current cellular processes. This approach involves the targeted selection of EVs using antibodies against the cell‐type‐specific surface marker(s) (Hamlett et al. 2018). EVs as cell‐based carriers: Mounting evidence suggests that secreted EVs may act as carriers of a variety of proteins and immune markers, potentially triggering or aggravating pathogenic processes by fusing with recipient cells, including neurons (Lin et al. 2015).

NDEVs are essential mediators of intercellular communication in the central nervous system (CNS), playing a critical role in neurodevelopmental processes, such as synaptic formation, neuronal differentiation, and plasticity (Li, Zhu et al. 2023). It has been noted that numerous cells in the CNS, including neurons, oligodendrocytes, and astrocytes, release EVs into the intercellular environment for communication (Huo et al. 2021). Alterations in the composition and secretion of NDEVs have been implicated in aging, contributing to a decline in neuroplasticity and increasing vulnerability to neurodegenerative diseases (Marzola et al. 2023). Although early studies on EVs focused on their roles on cellular processes (Fruhbeis et al. 2013; Mathivanan 2010; Schiera et al. 2007; Shin et al. 2010), recent research emphasizes the significant contribution of NDEVs in disease pathogenesis, particularly in AD, due to their involvement in amyloid and tau spreading (Elsherbini et al. 2020; Gabrielli et al. 2022; Sardar Sinha et al. 2018). Furthermore, recent studies suggest that NDEVs may hold therapeutic potential, as they can modulate neuroinflammation and offer neuroprotective effects in both aging and neurodegenerative contexts (Li, Zhu et al. 2023).

NDEVs are secreted from neuron cells into extracellular fluids, allowing them to interact with other neuronal cells or transverse the blood–brain barrier (BBB) to peripheral blood. They own cell‐specific surface markers that indicate their cell of origin and share the same route for biogenesis, secretion, and uptake similar to EVs. A deeper understanding of these processes is important for employing them in disease diagnosis and therapy for AD, where clinical translation of molecular studies is highly needed. This review focuses on the role of NDEVs in intercellular communication and the transfer of materials related to neurological diseases, as well as their involvement in neurodevelopment and aging.

## 2 NDEVs in AD

Blood is an important source for NDEV isolation from AD patients and controls, with samples collected into EDTA‐containing tubes (Nogueras‐Ortiz et al. 2020). NDEVs are released from the plasma membrane through endosomal pathways, subsequently entering extracellular fluids within the CNS, infiltrating other neural cells, or passing through the BBB into blood. During this process, NDEVs gather a variety of neuronal proteins of cellular membranes and cytosolic components (Goetzl et al. 2020).

When compared to EVs produced from blood cells, the number of NDEVs in circulation is quite low. To overcome the difficulty of distinguishing NDEV from blood, a relatively large blood sample volume is usually required for NDEV isolation. Prior research on EV biomarkers employed antibodies directed against L1 cell adhesion molecule (L1CAM), a surface marker mostly produced by neurons but also by kidney, dermal, and peripheral lymphocyte cells, to isolate NDEVs. It is followed by incubation with blood or CSF EVs. These enrichment methods were first done and optimized by Goetzl group (Fiandaca et al. 2015) and Zhang group (Shi et al. 2014). Goetzl group used both L1CAM and neural cell adhesion molecule (NCAM) for the enrichment of the blood EVs of the AD and frontotemporal dementia (FTD) patients. This enrichment method also demonstrated that the specificity of L1CAM is higher than NCAM, as NCAM can also bind to natural killer cells and natural killer T cells (Fiandaca et al. 2015). Many studies that investigate therapeutic or diagnostic biomarkers for neurodegenerative diseases use L1CAM‐enriched EVs (Arioz et al. 2021; Goetzl et al. 2018; Winston et al. 2018). An alternative approach for NDEV separation involves the use of 2.8 m superparamagnetic beads in an immunoaffinity assay (Eitan et al. 2023; Rufino‐Ramos et al. 2022). Although non‐neuronal cells express L1CAM at relatively low levels, its presence in other organs raises questions about the origin and purity of NDEVs. From this point of view, in a very recent study, Eitan et al. (2023) used the combination of neuron‐specific antigens such as GAP43 (growth‐associated protein 43) and NLGN3 (neuroligin 3) for NDEV capture. Hence, the isolation of NDEVs using neuron‐specific antigens will come over the debate about NDEV isolation.

### 2.1 NDEVs in AD Pathogenesis

NDEVs play a vital role in the mediating of the crosstalk between neurons and other cells of CNS for the spreading of the AD pathogenesis (Huo et al. 2021; Yerrapragada and Bihl 2022). In addition to actual cell‐to‐cell contact and the release of soluble factors by the cells, microglia and neurons can communicate by bidirectionally releasing EVs, which allows for the exchange of a wide variety of biomolecules over long distances (Szepesi et al. 2018). Emerging research suggests that NDEVs serve as important selective carriers for neuron–neuron or neuron–glia interaction in the brain by transmitting genetic information, various bioactive proteins, lipids, and, pathogenesis factors/molecules (Song et al. 2020).

EVs generated from neurons have been the main spotlight of studies on cargos in aging persons with AD. EVs participate in amyloid precursor protein (APP) processing, which is important for AD neuropathology. After being broken down into peptides, APP is delivered to EVs from early endosomes, where it may then be released from the cells. EVs from neurons contain toxic amyloid peptides and tau, which they can transfer to nearby cells, to other brain regions, and to the circulatory system for the spreading of disease pathogenic factors. This suggests that NDEVs, which can be obtained from either plasma or CSF, can specifically evaluate important neuropathological processes in CNS neurons (Hamlett et al. 2018). Detailed information about amyloid peptides and tau spreading via EVs is explained in detail in the following sections.

#### 2.1.1 NDEVs in Aβ Spreading

In AD pathogenesis, NDEV‐mediated cell‐to‐cell interactions are also crucial. The hallmark of AD is the intracerebral build‐up of Aβ, which results in the formation of Aβ plaques. EVs isolated from 5xFAD mice brain tissue were administered to wild‐type (WT) mice brains, where they were taken by neurons of WT mice brains. Researchers observed that Aβ was transported via 5xFAD EVs, which resulted in EV mediated Aβ neurotoxicity in WT mice (Elsherbini et al. 2020). Like in vivo results, experiments have demonstrated that EVs from Alzheimer's patients were found to mediate Aβ transport. For example, Alzheimer's patients’ EVs have elevated quantities of Aβ oligomers, which can transfer to recipient neurons in culture. In another study to investigate the Aβ propagation, isolated and labeled (PKH67) EVs from AD brains were given to human induced pluripotent stem cells (hiPSCs) or differentiated SH‐SY5Y cells. Then, the transfer of Aβ via EVs was evaluated via confocal microscopy and cytotoxicity assay. In conclusion, neuronal toxicity of Aβ was observed in recipient cells, which may be proof of Aβ transfer among the neuron‐to‐neuron transfer (Sardar Sinha et al. 2018). In another study, it was shown that Aβ in the EVs of SKNSH‐SY5Y neuroblastoma cells and primary neuronal cells (rat primary cortical neurons) in culture are released to extracellular space. According to their findings, all APP metabolites have the ability to be secreted into the extracellular space via EVs for APP metabolism (Vingtdeux et al. 2007; Yuyama and Igarashi 2017). There is a possibility that glial cell–derived EVs participate in the propagation of Aβ. A recent study indicates that microglial EVs with Aβ can move along the surface of neurons, which can intensify and spread early synaptic dysfunction in AD across the entorhinal cortex and dentate gyrus in the mouse brain (Gabrielli et al. 2022).

NDEVs may also play a significant role in the clearance of Aβ. Yuyama et al. (2015) observed the existence of Aβ in the EVs of CSF in cynomolgus monkeys and APP transgenic mice. Furthermore, it was noted that Aβ levels of EVs from older animals were decreased in CSF. When they infused the NDEVs into transgenic APP mice, Aβ depositions were reduced in the same manner that was formerly observed in neuroblastoma‐derived EVs (Yuyama and Igarashi 2017).

#### 2.1.2 NDEVs in Tau Spreading

Tau is important for cell‐to‐cell interactions; tau propagation involves neighboring cells taking up the protein within their cells. It has been suggested that tau absorption may occur through direct membrane fusion, micropinocytosis, or clathrin‐mediated endocytosis (Calafate et al. 2016; Christianson and Belting 2014). Many researchers are concentrating on the creation of tau antibodies as immunotherapies to stop the cell‐to‐cell transmission of pathogenic tau (Chai et al. 2011; Yanamandra et al. 2013). Moreover, tau is released into the extracellular area upon neuronal death (Zhang, Cao et al. 2021). The soluble‐free form of tau protein can interact with M1/M3 muscarinic receptors, which are found in certain glial cells and neurons. Increased intracellular calcium may result from the uptake of tau by these receptors (Diaz‐Hernandez et al. 2010; Gomez‐Ramos et al. 2008). However, extracellular tau interacts with fractalkine receptors on microglia (Bolos et al. 2017; Perea et al. 2018), which could facilitate the spread of tau (Asai et al. 2015).

EVs that contain tau protein have been discovered in human biofluids from AD patients, and EV‐mediated tau release and trans‐synaptic transmission have also been documented (Guix et al. 2018; Wang et al. 2017). In the most recent study by Ruan et al. (2020), EVs were isolated from frozen human AD control brain tissue. These isolated EVs were then injected into the hippocampal regions of 18‐month‐old female WT mice. The mice that received EVs of AD exhibited higher levels of p‐tau than those that received control EVs. This implies that EV transfer to mouse brains leads to the accumulation of oligomeric and fibrillar tau in mouse brains (Ruan 2022). In vivo animal studies have also shown the involvement of EVs in the spread of tau. EVs isolated from the brains of WT and rTg4510 transgenic mice (containing human tau with the P301L mutation) were injected into human WT tau transgenic ALZ17 mice. Six months later, the acceleration in tau phosphorylation and the formation of seed NFTs were observed in the mouse brains (Baker et al. 2016).

#### 2.1.3 NDEVs in Non‐Coding RNA Spreading

Non‐coding RNAs as cargo have been studied due to their ability to affect the cell‐to‐cell interaction in brain; particularly, the ones that are found in brain EVs of AD patients have been shown to affect the microglia function, such as phagocytosis (Luo et al. 2023). Because its increase in AD patients has been reported in clinical studies, ncRNA miR‐21 is considered to be one of the “inflamma‐microRNAs (miRNAs)” (Liang and Wang 2021). With the intention of exploring the role of miRNAs in EV on neuron–microglia communication, SH‐SY5Y and SWE cells were co‐cultured with HMC3 human microglia cells. It was shown that microglia internalize EVs from both cell types. EVs from SWE cells are also shown to cause microglial activation, as can be seen with an increase in tumor necrosis factor‐alpha (TNF‐α) and HMGB1 levels (Fernandes et al. 2018). In the follow‐up study from the same group, IFN‐γ‐activated HMC3 cells were co‐cultured with EVs derived from SWE cells to see the effect on NDEVs on microglia subtypes (Garcia et al. 2022). In addition to confirming the miR‐124 increase in SWE cells, it was observed that microglia responded to increased miR‐124 levels by altering protein levels related to immune function and inflammation. In another in vitro study, miR‐21‐5p transferred to BV2 microglia via EVs from PC12. These EVs carrying miR‐21‐5p were phagocytosed by microglia, leading to upregulated expression of miR‐21‐5p and subsequent M1 microglia polarization. The M1 polarization microglia had various effects, including the release of neuroinflammatory factors, inhibition of neurite outgrowth, increased accumulation of p‐tau, and PC12 cell death (Yin et al. 2020). A similar study was performed for miR‐124‐3p both in vivo and in vitro. According to the findings, M1 microglia and A1 astrocyte activation both in vivo and in vitro were suppressed by NDEVs, promoting functional behavioral recovery (Jiang et al. 2020).

### 2.2 NDEVs as Biomarkers for AD

Cargo materials that are carried by EVs can give insights into the biological changes in host cells. Therefore, EV contents are promising materials for biomarker research for many diseases, especially diseases like AD, where taking samples from the brain is difficult for diagnosis and treatment (Kulichikhin et al. 2021). In addition to their ability to forecast the beginning of dementia, biomarkers also dynamically correlate with cognition, enabling us to track patient reactions to care and assess brain recovery. For brain‐related diseases such as neurodegenerative diseases, EVs can be isolated from blood/plasma. These biomarkers have the ease of being relatively non‐invasive and able to continuously monitor the health of neurons and other types of brain cells (Gunes et al. 2022).

Several hypotheses for using NDEV contents as biomarkers, supported by experimental evidence, include the following: First, that NDEVs can cross the BBB; second, that only NDEVs contain neuron‐specific surface proteins; third, that NDEV cargo reflects the cell of NDEV origin; and, finally, that NDEV cargo is minimally modified after NDEVs are released.

#### 2.2.1 Protein Biomarkers

##### 2.2.1.1 Common Biomarkers Such As Aβ and Tau

Different NDEV contents were investigated as biomarkers for disease progression. Aβ (Zhao et al. 2020) or different types of tau (Fiandaca et al. 2015; Winston et al. 2016) are commonly used AD biomarkers. Because of the invasive nature of taking CSF samples, studies have been done on the change of those molecules in plasma NDEVs as an alternative source. EV contents isolated from blood samples of mild cognitive impairment (MCI), AD, or FTD patients have been compared with their CSF contents or CSF contents that have been analyzed before. In many studies, it was shown that changes in AD‐related molecule levels in EVs reflect the change in the CSF, and it is consistent in the follow‐up tests as well (Goetzl et al. 2016; Jia et al. 2019; Li et al. 2022). Significant changes in protein levels between patients that experience different dementia types have been shown to contribute to the differential diagnosis of AD. For example, discriminant modeling generated using p‐T181‐tau, p‐S396‐tau, and Aβ_(1–42)_ from the data obtained from plasma‐isolated NDEVs yielded 96.4% accuracy for AD patients and 87.5% accuracy for FTD patients for disease prediction. This model could also predict the disease progression 10 years before disease onset (Fiandaca et al. 2015). Protein biomarkers found in NDEVs can help to predict the progression of MCI to AD, especially when used with other diagnostic methods such as the evaluation of olfactory functions with sniffing sticks. An increase in Aβ levels in NDEVs and a decrease in olfactory function have been shown to enhance the accuracy of predicting the conversion from MCI to AD (Zhao et al. 2020). Aβ_(1–42)_, p‐tau, and total tau (t‐tau) have already been shown to increase in the CSF of AD patients or MCI patients that were converted to AD (Wallin et al. 2006).

Boyer et al. conducted a study in 2024: The levels and composition of plasma NDEVs, specifically Aβ40, Aβ42, and tau, were compared between CN (cognitively normal) individuals and AD patients. The quantity of plasma NDEVs remained stable with age and did not differ significantly between individuals with AD and CN. Notably, in CN participants, there was a negative correlation between the levels of soluble biomarkers and the number of NDEVs. In contrast, this correlation was absent in patients with AD, indicating a potential alteration in the mechanisms governing the production and release of these biomarkers under pathological conditions (Boyer et al. 2024). In another study that compares Aβ and p‐tau levels between healthy controls and AD patients, Singh et al. (2024) demonstrated that the concentration of EVs is significantly greater in individuals with AD than in healthy control subjects. Notably, the expression of Aβ_(1–42)_ within these EVs is markedly increased in AD cases compared to healthy controls. Additionally, the levels of p‐tau derived from EVs are also found to be elevated in the AD group relative to the control group. Conversely, the expression of synaptophysin in EVs is significantly reduced in the AD cohort when compared to healthy controls.

##### 2.2.1.2 Synaptic Proteins

**FIGURE 1:**
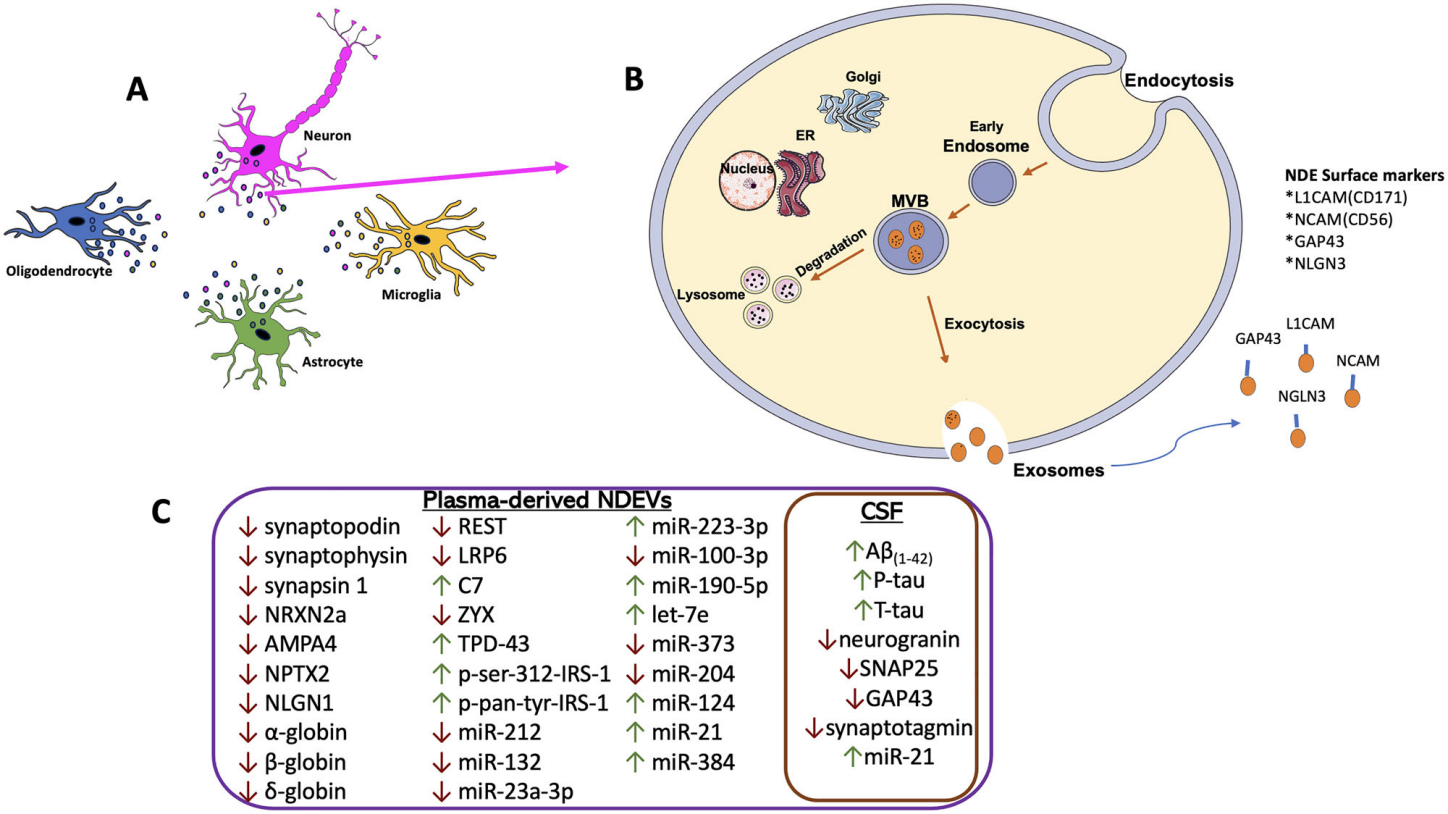
(A) NDEVs in cell‐to‐cell interaction; (B) neuronal extracellular vesicle biogenesis and secretion, including surface markers that are used for NDEV isolation; (C) summary of potential biomarkers for AD found in NDEVs or CSF. CSF, cerebrospinal fluid; GAP43, growth‐associated protein 43; L1CAM, L1 cell adhesion molecule; NCAM, neural cell adhesion molecule; NDEV, neuron‐derived extracellular vesicle; NLGN3, Neuroligin 3. *Source*: The figure was partly generated using Servier Medical Art, provided by Servier, licensed under a Creative Commons Attribution 3.0 unported license.

Association of synaptic protein levels with AD has been an area of interest because of the association of AD with neuron loss and decreased synaptic plasticity. Previously, it was shown that synaptic proteins such as GAP43, neurogranin (NRGN), synaptotagmin, and SNAP25 had been decreased in CSF samples and plasma‐isolated NDEVs of AD patients (Agliardi et al. 2019; Goetzl et al. 2016; Jia et al. 2021; Winston et al. 2018) (Figure 1). In one of the first studies that evaluated SNAP‐25 levels in serum, SNAP‐25 levels in serum NDEVs from AD patients were demonstrated to be significantly lower than in NDEVs from their age‐matched controls. This decrease was also correlated with the mini‐mental state examination (MMSE) scores of patients and healthy controls, showing its potential to be a biomarker for synaptic degradation (Agliardi et al. 2019). Studies with plasma NDEVs have also shown similar results where NRGN, synaptophysin, synaptotagmin, and synaptopodin were significantly lower in MCI and AD patient samples (Winston et al. 2018).

Alternatively, synaptic protein levels in NDEVs can be used as a differential diagnosis. GAP43 and Synapsin 1 were reduced only in AD patient NDEVs and not in FTD patient EVs (Goetzl et al. 2016). Another study (Goetzl et al. 2018) demonstrates that synaptic proteins like Neurexin 2a (NRXN2a), AMPA4, Neuronal pentraxin 2 (NPTX2), and Neuroligin 1 (NLGN1) have been decreased with disease progression. Both of those studies started collecting samples in pre‐clinical stages, approximately 7–8 years before diagnosis, and showed the correlation of cognitive decline with exosomal proteins, which further supports the idea of proteins in NDEVs as biomarkers for early diagnosis of AD.

##### 2.2.1.3 Transcription Factors

Transcription factors (TFs) like REST have been known to protect neurons from stress. A decrease in such TFs has been associated with neurodegenerative diseases, including AD and MCI (Lu et al. 2014). Therefore, TFs and proteins that are involved in their expression are presented as possible biomarkers for diagnosis or differential diagnosis of AD. Studies have shown that REST and LRP6 concentrations in NDEVs isolated from AD patients have been significantly lower than in EVs isolated from healthy controls (Goetzl et al. 2015). A decrease in REST could only be seen in AD patients or MCI patients that are converted to AD but not in stable MCI patients, which indicates that REST in neuronal EVs can be used as a differential diagnostic biomarker for AD (Winston et al. 2016).

##### 2.2.1.4 Other Protein Biomarkers

TDP‐43, TAR DNA binding protein of 43 kDa, has been shown to be abruptly phosphorylated in many neurodegenerative disorders, including AD. From its correlation with cognitive decline, the relationship of TDP‐43 with disease severity in AD was shown in many studies, therefore suggesting its potential as a biomarker (Josephs et al. 2014; Meneses et al. 2021). Because of the low levels in CSF, total length TDP‐43 levels were examined in plasma NDEVs. Enzyme‐linked immunosorbent assay (ELISA) results of TDP‐43 were shown to be significantly increased in AD patients when compared to healthy controls (Zhang et al. 2020). There was no relationship found between disease severity and TDP‐43 levels, possibly due to not including p‐TDP43. However, these results still exhibit a candidate protein to traditional AD protein biomarkers.

Biomarkers for other diseases, such as p‐serine‐312‐IRS1 and p‐pan‐tyrosine‐IRS1 for diabetes mellitus (DM), can also be used for the diagnosis of AD. Because insulin resistance affects similar brain regions in both diseases, molecules that potentially cause this insulin resistance can be promising biomarkers. In one of the first studies that investigated the content of NDEVs as biomarkers, it was shown that p‐serine 312‐IRS‐1 and p‐pan‐tyrosine‐IRS1 were found in AD patients’ NDEVs and were significantly higher than in DM2 or FTD patient EVs. In addition, p‐serine 312‐IRS‐1 and p‐pan‐tyrosine‐IRS1 were shown to be different 1 and 10 years before diagnosis, indicating p‐serine 312‐IRS‐1 and p‐pan‐tyrosine‐IRS1 molecules can be used as differential diagnoses and as predictive biomarkers for AD (Kapogiannis et al. 2015).

##### 2.2.1.5 Proteomics Studies

Proteomics studies can reveal protein biomarkers that can be useful for disease diagnosis, differential diagnosis, progression, and predicting response to treatment. Although it is not cost‐ or time‐efficient, proteomic analysis in the context of diagnosis and biomarker studies can be revealed through profiling of biological samples. Proteome profiling of patient and healthy control samples performed by our group demonstrated that hemoglobin subunits alpha‐, beta‐, and delta‐globin were significantly increased in AD patients (Arioz et al. 2021). Serum EVs from around 20 AD patients and 23 age‐matched controls were isolated and analyzed. GO analysis of EV‐derived proteins not only showed peptides belonging to the hemoglobin family but also showed oxygen transport and cellular oxygen detoxification as dysregulated pathways. The findings suggest hemoglobin as an alternative biomarker to conventional protein biomarkers of AD, and it can contribute to inflammation caused by reactive oxygen species. Different proteomic studies suggest more biomarkers that can be used both in the diagnosis of AD, such as Complement component 7 (C7) and zyxin (ZYX), and in differential diagnosis from MCI, such as apolipoprotein A‐II (APOA2) (Zhong et al. 2021).

#### 2.2.2 miRNAs

Apart from proteins, non‐coding RNAs, such as miRNAs, have the potential to serve as biomarkers. A study by Cha et al. investigates the miRNA content of EVs using miRNA arrays. For the determination of miRNAs that will be assessed in NDEVs, brain tissues from AD patients, high‐pathological controls that had amyloid plaques present in their brains but did not have cognitive impairments at the time of death, and pathology‐free controls were examined for the miRNA levels that have the highest fold change between groups. The results suggest miR‐212 and miR‐132 as potential biomarkers for AD because they were decreased the most in AD EVs, and they mostly play roles in neuron maturation, survival, and plasticity. Among four of the miRNAs that were chosen, miR‐212 and miR‐132 have been determined to be promising for diagnosis (Cha et al. 2019). Different studies that use arrays suggest various miRNA biomarkers that are part of AD‐related pathways. MiR‐23a‐3p, miR‐223‐3p, miR‐100‐3p, and miR‐190‐5p were shown to be altered in NDEVs of AD patients compared to controls. It was also one of the first studies that compared total plasma EVs with NDEVs, proposing a specific miRNA signature of NDEVs different from total peripheral EVs (Serpente et al. 2020). miR‐384 has also been suggested as a biomarker for AD diagnosis, based on results observed in mice studies. To test this hypothesis in humans, Li et al. (2022) examined the miR‐384 levels in NCAM‐labeled EVs from patient plasma and observed the significantly increased levels of miR‐384 levels in amnestic MCI and AD patients when compared to other patients with cognitive dysfunction and controls.

Altered levels of miRNAs in NDEVs can also be profiled by next‐generation sequencing (NGS). In their study, Pounders et al. have sequenced RNA with NGS from healthy controls and FTD and AD patients to reveal that miR‐122 and miR‐3591 have been decreased in AD samples compared to FTD. These results suggest that miRNA profiling of NDEVs can be promising platforms for differential diagnosis (Pounders et al. 2022). Another investigation of miRNA content of small NDEVs done with NGS by our group, contrarily, has shown that let‐7e levels in AD EVs were significantly higher than in healthy control EVs. We also investigated the effect of neuron–microglia interactions and found that EVs with high let‐7e amounts increase inflammation in microglial cells. The results suggest let‐7e as a diagnostic biomarker and a contributing factor to the pathogenesis of AD (Durur et al. 2022).

miRNAs whose levels were altered in disease conditions can be involved in different pathways. For example, miR‐373 has been shown to be upregulated in patients with HSV‐1 infection and where immune activation is suppressed (Xie et al. 2018). Considering the immune system involvement in AD, Taşdelen et al. (2022) investigated altered levels of miR‐373 and miR‐204, miRNAs that are known to be involved in NLRP3 inflammasome activation. NLRP3 inflammasome is an important component of the immune system, and the abnormal activation of NLRP3 inflammasome causes neurodegeneration in AD (Kelley et al. 2019). Two of the NLRP3 inflammasome inducers, P2X purinoceptor 7 (P2RX7) and thioredoxin‐interacting protein (TXNIP), have been shown to be related with miR‐373 (Zhang et al. 2018) and miR‐204 (Xu et al. 2013), respectively. On the basis of this knowledge, their study on miR‐373 and miR‐204 levels in NDEVs demonstrated that both miRNA levels were decreased significantly in NDEVs from AD patients. This result was contradicted with the literature, where CSF levels of miR‐373 were not changed between patient and control groups, which presented the importance of information that is carried out by EVs and the role of EVs in biomarker studies (Tasdelen et al. 2022).

## 3 NDEVs in Aging

Aging is defined by a gradual decline in the physiological functions of tissues and organs. Recent research indicates that NDEVs play a significant role in the aging process (Manni et al. 2023). It was proposed that a decrease in adult hypothalamic neurogenesis has physiological significance in systemic aging (Zhang et al. 2013). Additionally, the authors showed that hypothalamic NDEVs contributed to a pool of CSF miRNAs with their cargo in addition to modulating the local neurogenic niche (miR‐335‐3p, miR‐192) (Bonetto and Grilli 2023; Zhang et al. 2017). Certain miRNAs seemed to be downregulated in NDEVs released during aging (miR‐466m‐5p, miR‐483‐5p, miR‐3072‐5p, miR‐7046‐5p). Compared to mice treated with a vehicle, middle‐aged mice whose third ventricle was injected with NDEVs of postnatal cultivated hypothalamic NSCs showed enhanced locomotion, coordination, cognitive function, and sociality, as well as a reversal of age‐related physiological decline (Zhang et al. 2017).

NGS of RNAs in human NPC‐derived NDEVs has shed light on the intricate behaviors of these cargos. This research has pinpointed specific miRNAs in EVs that exhibit differential expression when compared to their parent cells, implicating them in processes related to aging and neural plasticity. Stevanato et al. (2016) identified a distinct array of miRNAs that are more prevalent in exosomal preparations than in cellular ones, derived from a clinical‐grade human NSC clonal line. Their findings revealed several miRNAs that are differentially expressed between hNSCs and hNSC‐derived NDEVs, with hsa‐miR‐1246, hsa‐miR‐4488, hsa‐miR‐4508, hsa‐miR‐4492, and hsa‐miR‐4516 being the most significantly enriched in EVs (Bonetto and Grilli 2023; Stevanato et al. 2016). These findings highlight the therapeutic potential of NDEVs in counteracting age‐related physiological decline through the modulation of miRNA expression and neurogenic activity.

## 4 NDEVs in Neurodevelopment

NDEVs play significant autocrine and paracrine roles in neurogenesis, influencing neurodevelopment (Losurdo and Grilli 2020; Sharma et al. 2013). NDEVs from NSC and NPC play a crucial role in determining cell fate, facilitating the commitment to neuronal lineages. Cultured neural stem and progenitor cells (NSPCs) generate and release NDEVs in both proliferative and differentiative states, potentially impacting various facets of neurogenesis. Notably, NDEVs from differentiated mouse embryonic NSPCs have been shown to induce differentiation in proliferating NSPCs (Stronati et al. 2019). Proteomic analyses of NDEVs produced during the quiescent and proliferative phases of NSCs have demonstrated their role in maintaining quiescence and facilitating the transition of NSCs between proliferation and quiescence (Zhang, Rong et al. 2021). Furthermore, the research indicated that quiescent NSCs (qNSCs) may utilize NDEVs to eliminate specific proteins, including ribosomal proteins (which are abundant in qNSC‐derived NDEVs), to regulate quiescence and sustain low levels of protein translation. Notably, PCSK6 (Anacker and Hen 2017), a convertase that enhances cell proliferation through the ERK1/2 and STAT3 signaling pathways, was found to be enriched in activated NSC‐derived NDEVs (Yuan et al. 2021).

Most existing literature is based on in vitro research rather than in vivo EV administration models. Although there are few in vivo studies on NDEVs, it has been demonstrated that NDEVs isolated from induced pluripotent stem cell (iPSC)‐derived NSCs and administered to rodents play a significant role in neurodevelopment and in the regulation of neurogenesis in the adult hippocampus (Cossetti et al. 2014; Gharbi et al. 2023; Sullivan et al. 2015). Furthermore, NDEVs from human iPSC‐derived NSCs exhibited an increased presence of proteins like Agrin and pentraxin‐3, which have been previously identified as significant contributors to neurogenesis in the adult hippocampus (Rodriguez‐Grande et al. 2015; Zhang et al. 2020). Collectively, these compelling findings may assist in identifying potential candidates among the proteins associated with activated NSC‐derived NDEVs that could reactivate NSCs and mitigate age‐ or pathology‐related dysfunctions within neurogenic niches (Bonetto and Grilli 2023).

miRNAs have recently gained particular attention, as a growing body of evidence indicates that specific miRNAs, encapsulated in EVs, are associated with neurodevelopment and neurogenesis. For instance, NDEVs from NSCs can deliver miRNAs, including miR‐124, to target cells, thereby facilitating neuronal differentiation (Ma et al. 2024). Moreover, miR‐9, which is significantly expressed in NDEVs derived from primary embryonic NSCs, promotes the in vitro differentiation and maturation of NSCs into neuronal lineages by modulating Hes‐1 expression (Yuan et al. 2021). A subsequent investigation revealed that NDEVs from NPCs are capable of promoting the differentiation of cortical NPCs into neuronal cells when cultured in differentiation medium, whereas iNPC‐derived NDEVs exhibited significantly reduced efficacy in facilitating this differentiation process (Ma et al. 2019). Microarray analysis indicated that NDEVs from NPCs contain significantly higher levels of miRNA‐21a compared to iNPC‐derived NDEVs. The application of a miRNA‐21a mimic and a miRNA‐21a inhibitor demonstrated that the mimic reduced the percentage of GFAP^+^ and tuj1^+^ glial cells while promoting the development of neuronal cells, whereas the inhibitor produced contrary effects. Overall, these findings underscore the influence of miRNA‐21a in steering the fate of NPCs toward neurogenesis instead of gliogenesis (Stronati et al. 2019). Moreover, in another study, it was demonstrated that NDEVs derived from NSCs in neonatal subventricular zone (SVZ) possess the ability to modulate microglial activity and are enriched with miRNAs such as miR‐9, let‐7, and miR‐26, which influence microglial morphology (Gamage and Fraser 2021; Morton et al. 2018; Yao et al. 2014). Transfection of NDEVs with synthetic let‐7 miRNA altered microglial morphology and suppressed NSC proliferation in SVZ (Kumar et al. 2015; Lehmann et al. 2012). The researchers proposed that NDEV release regulates the communication network between NSCs and microglia, thereby influencing NSC proliferation in SVZ (Zhang et al. 2015). To sum up, miR‐21a, miR‐9, miR‐let‐7, and miR‐124 are commonly identified miRNAs in NDEVs from NPCs during neurodevelopment and neurogenesis. These miRNAs play important roles in proliferation, differentiation, maturation, and fate specification (Bonetto and Grilli 2023; Ma et al. 2024; Park et al. 2022; Parkash and Radhakrishna 1987; Yang et al. 2018).

## 5 Conclusion

The role of specific cell‐derived EVs in the diagnosis, monitoring, and treatment of AD arouses great interest. But the results of the different studies are inconsistent, and many mechanisms are still not clarified. Along with NDEVs, EVs released from other cell types in CNS can also have significant effects on AD pathology. Such studies also assist with disease biomarkers for early and differential diagnosis. Hence, the process and mechanism of specific cell‐derived EVs for their origin, formation, and transportation in their exact roles in AD need to be clarified. In addition, extraction, isolation, and characterization in the aspects of size and number of specific cells derived from EVs are still unclear and need to be optimized. Although many studies used L1CAM as a therapeutic or diagnostic marker for the enrichment of NDEVs in neurodegenerative diseases (Arioz et al. 2021; Goetzl et al. 2018; Nam et al. 2020; Winston et al. 2018), there is a controversial approach to using L1CAM. It is important to establish a consensus on this issue in order to facilitate the translation of the studies into clinical practice.

Overall, EVs are emerging as key mediators of intercellular communication throughout neurodevelopment and aging, regulating essential processes, such as synaptic plasticity, myelination, and neuroinflammation. Age‐associated changes in NDEV composition and secretion may underlie impaired neurogenesis and contribute to the pathogenesis of neurodegenerative diseases such as AD. Continued research into the molecular mechanisms governing NDEV biology will be critical to unlocking their full potential in understanding and treating AD.

## Author Contributions

Ceren Perihan Gonul, Bilge Karacicek, and Sermin Genc wrote the first draft of the manuscript. Sermin Genc revised and approved the submitted version of the manuscript. All authors contributed to the article and approved the submitted version.

## Conflicts of Interest

The authors declare no conflicts of interest.

## Data Availability

The data used are available within the article and can be obtained from the corresponding author.

## References

[dneu22984-bib-0001] Agliardi, C. , F. R. Guerini , M. Zanzottera , A. Bianchi , R. Nemni , and M. Clerici . 2019. “SNAP‐25 in Serum Is Carried by Exosomes of Neuronal Origin and Is a Potential Biomarker of Alzheimer's Disease.” Molecular Neurobiology 56, no. 8: 5792–5798. 10.1007/s12035-019-1501-x. 30680692

[dneu22984-bib-0002] Alawode, D. O. T. , A. J. Heslegrave , N. J. Ashton , et al. 2021. “Transitioning From Cerebrospinal Fluid to Blood Tests to Facilitate Diagnosis and Disease Monitoring in Alzheimer's Disease.” Journal of Internal Medicine 290, no. 3: 583–601. 10.1111/joim.13332. 34021943 PMC8416781

[dneu22984-bib-0003] American Psychiatric Associaction . 2013. Diagnostic and Statistical Manual of Mental Disorders. 5th ed. American Psychiatric Associaction.

[dneu22984-bib-0004] Anacker, C. , and R. Hen . 2017. “Adult Hippocampal Neurogenesis and Cognitive Flexibility—Linking Memory and Mood.” Nature Reviews Neuroscience 18, no. 6: 335–346. 10.1038/nrn.2017.45. 28469276 PMC6261347

[dneu22984-bib-0005] Arioz, B. I. , K. U. Tufekci , M. Olcum , et al. 2021. “Proteome Profiling of Neuron‐Derived Exosomes in Alzheimer's Disease Reveals Hemoglobin as a Potential Biomarker.” Neuroscience Letters 755: 135914. 10.1016/j.neulet.2021.135914. 33901610

[dneu22984-bib-0006] Asai, H. , S. Ikezu , S. Tsunoda , et al. 2015. “Depletion of Microglia and Inhibition of Exosome Synthesis Halt Tau Propagation.” Nature Neuroscience 18, no. 11: 1584–1593. 10.1038/nn.4132. 26436904 PMC4694577

[dneu22984-bib-0007] Baker, S. , J. C. Polanco , and J. Gotz . 2016. “Extracellular Vesicles Containing P301L Mutant Tau Accelerate Pathological Tau Phosphorylation and Oligomer Formation But Do Not Seed Mature Neurofibrillary Tangles in ALZ17 Mice.” Journal of Alzheimer's Disease 54, no. 3: 1207–1217. 10.3233/JAD-160371. 27567840

[dneu22984-bib-0008] Bolos, M. , M. Llorens‐Martin , J. R. Perea , et al. 2017. “Absence of CX3CR1 Impairs the Internalization of Tau by Microglia.” Molecular Neurodegeneration 12, no. 1: 59. 10.1186/s13024-017-0200-1. 28810892 PMC5558740

[dneu22984-bib-0009] Bonetto, V. , and M. Grilli . 2023. “Neural Stem Cell‐Derived Extracellular Vesicles: Mini Players With Key Roles in Neurogenesis, Immunomodulation, Neuroprotection and Aging.” Frontiers in Molecular Biosciences 10: 1187263. 10.3389/fmolb.2023.1187263. 37228583 PMC10203560

[dneu22984-bib-0010] Boyer, E. , L. Deltenre , M. Dourte , et al. 2024. “Comparison of Plasma Soluble and Extracellular Vesicles‐Associated Biomarkers in Alzheimer's Disease Patients and Cognitively Normal Individuals.” Alzheimer's Research & Therapy 16, no. 1: 141. 10.1186/s13195-024-01508-6. PMC11212434 38943196

[dneu22984-bib-0011] Calafate, S. , W. Flavin , P. Verstreken , and D. Moechars . 2016. “Loss of Bin1 Promotes the Propagation of Tau Pathology.” Cell Reports 17, no. 4: 931–940. 10.1016/j.celrep.2016.09.063. 27760323

[dneu22984-bib-0012] Cha, D. J. , D. Mengel , M. Mustapic , et al. 2019. “miR‐212 and miR‐132 Are Downregulated in Neurally Derived Plasma Exosomes of Alzheimer's Patients.” Frontiers in Neuroscience 13: 1208. 10.3389/fnins.2019.01208. 31849573 PMC6902042

[dneu22984-bib-0013] Chai, X. , S. Wu , T. K. Murray , et al. 2011. “Passive Immunization With Anti‐Tau Antibodies in Two Transgenic Models: Reduction of Tau Pathology and Delay of Disease Progression.” Journal of Biological Chemistry 286, no. 39: 34457–34467. 10.1074/jbc.M111.229633. 21841002 PMC3190817

[dneu22984-bib-0014] Christianson, H. C. , and M. Belting . 2014. “Heparan Sulfate Proteoglycan as a Cell‐Surface Endocytosis Receptor.” Matrix Biology 35: 51–55. 10.1016/j.matbio.2013.10.004. 24145152

[dneu22984-bib-0015] Cossetti, C. , N. Iraci , T. R. Mercer , et al. 2014. “Extracellular Vesicles From Neural Stem Cells Transfer IFN‐Gamma Via Ifngr1 to Activate Stat1 Signaling in Target Cells.” Molecular Cell 56, no. 2: 193–204. 10.1016/j.molcel.2014.08.020. 25242146 PMC4578249

[dneu22984-bib-0016] Diaz‐Hernandez, M. , A. Gomez‐Ramos , A. Rubio , et al. 2010. “Tissue‐Nonspecific Alkaline Phosphatase Promotes the Neurotoxicity Effect of Extracellular Tau.” Journal of Biological Chemistry 285, no. 42: 32539–32548. 10.1074/jbc.M110.145003. 20634292 PMC2952256

[dneu22984-bib-0017] Dong, X. , D. Zheng , and J. Nao . 2020. “Circulating Exosome microRNAs as Diagnostic Biomarkers of Dementia.” Frontiers in Aging Neuroscience 12: 580199. 10.3389/fnagi.2020.580199. 33093831 PMC7506134

[dneu22984-bib-0018] Durur, D. Y. , B. Tastan , K. Ugur Tufekci , et al. 2022. “Alteration of miRNAs in Small Neuron‐Derived Extracellular Vesicles of Alzheimer's Disease Patients and the Effect of Extracellular Vesicles on Microglial Immune Responses.” Journal of Molecular Neuroscience 72, no. 6: 1182–1194. 10.1007/s12031-022-02012-y. 35488079

[dneu22984-bib-0019] Eitan, E. , T. Thornton‐Wells , K. Elgart , et al. 2023. “Synaptic Proteins in Neuron‐Derived Extracellular Vesicles as Biomarkers for Alzheimer's Disease: Novel Methodology and Clinical Proof of Concept.” Extracellular Vesicles and Circulating Nucleic Acids 4, no. 1: 133–150. 10.20517/evcna.2023.13. 37842184 PMC10568955

[dneu22984-bib-0020] Elsherbini, A. , H. Qin , Z. Zhu , P. Tripathi , S. M. Crivelli , and E. Bieberich . 2020. “In Vivo Evidence of Exosome‐Mediated Abeta Neurotoxicity.” Acta Neuropathologica Communications 8, no. 1: 100. 10.1186/s40478-020-00981-y. 32631455 PMC7339450

[dneu22984-bib-0021] Fernandes, A. , A. R. Ribeiro , M. Monteiro , G. Garcia , A. R. Vaz , and D. Brites . 2018. “Secretome From SH‐SY5Y APPSwe Cells Trigger Time‐Dependent CHME3 Microglia Activation Phenotypes, Ultimately Leading to miR‐21 Exosome Shuttling.” Biochimie 155: 67–82. 10.1016/j.biochi.2018.05.015. 29857185

[dneu22984-bib-0022] Fiandaca, M. S. , D. Kapogiannis , M. Mapstone , et al. 2015. “Identification of Preclinical Alzheimer's Disease by a Profile of Pathogenic Proteins in Neurally Derived Blood Exosomes: A Case‐Control Study.” Alzheimer's & Dementia 11, no. 6: 600–607. e601. 10.1016/j.jalz.2014.06.008. PMC4329112 25130657

[dneu22984-bib-0023] Frisoni, G. B. , N. C. Fox , C. R. Jack Jr. , P. Scheltens , and P. M. Thompson . 2010. “The Clinical Use of Structural MRI in Alzheimer Disease.” Nature reviews Neurology 6, no. 2: 67–77. 10.1038/nrneurol.2009.215. 20139996 PMC2938772

[dneu22984-bib-0024] Fruhbeis, C. , D. Frohlich , W. P. Kuo , et al. 2013. “Neurotransmitter‐Triggered Transfer of Exosomes Mediates Oligodendrocyte‐neuron Communication.” PLoS Biology 11, no. 7: e1001604. 10.1371/journal.pbio.1001604. 23874151 PMC3706306

[dneu22984-bib-0025] Gabrielli, M. , I. Prada , P. Joshi , et al. 2022. “Microglial Large Extracellular Vesicles Propagate Early Synaptic Dysfunction in Alzheimer's Disease.” Brain 145, no. 8: 2849–2868. 10.1093/brain/awac083. 35254410 PMC9420022

[dneu22984-bib-0026] Gamage, T. , and M. Fraser . 2021. “The Role of Extracellular Vesicles in the Developing Brain: Current Perspective and Promising Source of Biomarkers and Therapy for Perinatal Brain Injury.” Frontiers in Neuroscience 15: 744840. 10.3389/fnins.2021.744840. 34630028 PMC8498217

[dneu22984-bib-0027] Garcia, G. , A. Fernandes , F. Stein , and D. Brites . 2022. “Protective Signature of IFNgamma‐Stimulated Microglia Relies on miR‐124‐3p Regulation From the Secretome Released by Mutant APP Swedish Neuronal Cells.” Frontiers in Pharmacology 13: 833066. 10.3389/fphar.2022.833066. 35620289 PMC9127204

[dneu22984-bib-0028] Gharbi, T. , C. Liu , H. Khan , Z. Zhang , G. Y. Yang , and Y. Tang . 2023. “Hypoxic Preconditioned Neural Stem Cell‐Derived Extracellular Vesicles Contain Distinct Protein Cargo From Their Normal Counterparts.” Current Issues in Molecular Biology 45, no. 3: 1982–1997. 10.3390/cimb45030127. 36975497 PMC10047917

[dneu22984-bib-0029] Goetzl, E. J. , E. L. Abner , G. A. Jicha , D. Kapogiannis , and J. B. Schwartz . 2018. “Declining Levels of Functionally Specialized Synaptic Proteins in Plasma Neuronal Exosomes With Progression of Alzheimer's Disease.” FASEB Journal 32, no. 2: 888–893. 10.1096/fj.201700731R. 29025866 PMC5888398

[dneu22984-bib-0030] Goetzl, E. J. , A. Boxer , J. B. Schwartz , et al. 2015. “Low Neural Exosomal Levels of Cellular Survival Factors in Alzheimer's Disease.” Annals of Clinical and Translational Neurology 2, no. 7: 769–773. 10.1002/acn3.211. 26273689 PMC4531059

[dneu22984-bib-0031] Goetzl, E. J. , D. Kapogiannis , J. B. Schwartz , et al. 2016. “Decreased Synaptic Proteins in Neuronal Exosomes of Frontotemporal Dementia and Alzheimer's Disease.” FASEB Journal 30, no. 12: 4141–4148. 10.1096/fj.201600816R. 27601437 PMC5102122

[dneu22984-bib-0032] Goetzl, E. J. , C. B. Peltz , M. Mustapic , D. Kapogiannis , and K. Yaffe . 2020. “Neuron‐Derived Plasma Exosome Proteins After Remote Traumatic Brain Injury.” Journal of Neurotrauma 37, no. 2: 382–388. 10.1089/neu.2019.6711. 31441374 PMC6964810

[dneu22984-bib-0033] Gomez‐Ramos, A. , M. Diaz‐Hernandez , A. Rubio , M. T. Miras‐Portugal , and J. Avila . 2008. “Extracellular Tau Promotes Intracellular Calcium Increase Through M1 and M3 Muscarinic Receptors in Neuronal Cells.” Molecular and Cellular Neuroscience 37, no. 4: 673–681. 10.1016/j.mcn.2007.12.010. 18272392

[dneu22984-bib-0034] Guix, F. X. , G. T. Corbett , D. J. Cha , et al. 2018. “Detection of Aggregation‐Competent Tau in Neuron‐Derived Extracellular Vesicles.” International Journal of Molecular Sciences 19, no. 3: 663. 10.3390/ijms19030663. 29495441 PMC5877524

[dneu22984-bib-0035] Gunes, S. , Y. Aizawa , T. Sugashi , M. Sugimoto , and P. P. Rodrigues . 2022. “Biomarkers for Alzheimer's Disease in the Current State: A Narrative Review.” International Journal of Molecular Sciences 23, no. 9: 4962. 10.3390/ijms23094962. 35563350 PMC9102515

[dneu22984-bib-0036] Hamlett, E. D. , A. Ledreux , H. Potter , et al. 2018. “Exosomal Biomarkers in Down Syndrome and Alzheimer's Disease.” Free Radical Biology and Medicine 114: 110–121. 10.1016/j.freeradbiomed.2017.08.028. 28882786 PMC6135098

[dneu22984-bib-0037] Hampel, H. , and K. Blennow . 2004. “CSF Tau and Beta‐Amyloid as Biomarkers for Mild Cognitive Impairment.” Dialogues in Clinical Neuroscience 6, no. 4: 379–390. https://www.ncbi.nlm.nih.gov/pubmed/22034251. 22034251 10.31887/DCNS.2004.6.4/hhampel PMC3181816

[dneu22984-bib-0038] Hansen, D. V. , J. E. Hanson , and M. Sheng . 2018. “Microglia in Alzheimer's Disease.” Journal of Cell Biology 217, no. 2: 459–472. 10.1083/jcb.201709069. 29196460 PMC5800817

[dneu22984-bib-0039] Huo, L. , X. Du , X. Li , S. Liu , and Y. Xu . 2021. “The Emerging Role of Neural Cell‐Derived Exosomes in Intercellular Communication in Health and Neurodegenerative Diseases.” Frontiers in Neuroscience 15: 738442. 10.3389/fnins.2021.738442. 34531720 PMC8438217

[dneu22984-bib-0040] Jia, L. , Q. Qiu , H. Zhang , et al. 2019. “Concordance Between the Assessment of Abeta42, T‐tau, and P‐T181‐tau in Peripheral Blood Neuronal‐Derived Exosomes and Cerebrospinal Fluid.” Alzheimer's & Dementia 15, no. 8: 1071–1080. 10.1016/j.jalz.2019.05.002. 31422798

[dneu22984-bib-0041] Jia, L. , M. Zhu , C. Kong , et al. 2021. “Blood Neuro‐Exosomal Synaptic Proteins Predict Alzheimer's Disease at the Asymptomatic Stage.” Alzheimer's & Dementia 17, no. 1: 49–60. 10.1002/alz.12166. PMC7984076 32776690

[dneu22984-bib-0042] Jiang, D. , F. Gong , X. Ge , et al. 2020. “Neuron‐Derived Exosomes‐Transmitted miR‐124‐3p Protect Traumatically Injured Spinal Cord by Suppressing the Activation of Neurotoxic Microglia and Astrocytes.” Journal of Nanobiotechnology 18, no. 1: 105. 10.1186/s12951-020-00665-8. 32711535 PMC7382861

[dneu22984-bib-0043] Josephs, K. A. , J. L. Whitwell , S. D. Weigand , et al. 2014. “TDP‐43 Is a Key Player in the Clinical Features Associated With Alzheimer's Disease.” Acta Neuropathologica 127, no. 6: 811–824. 10.1007/s00401-014-1269-z. 24659241 PMC4172544

[dneu22984-bib-0044] Kapogiannis, D. , A. Boxer , J. B. Schwartz , et al. 2015. “Dysfunctionally Phosphorylated Type 1 Insulin Receptor Substrate in Neural‐Derived Blood Exosomes of Preclinical Alzheimer's Disease.” FASEB Journal 29, no. 2: 589–596. 10.1096/fj.14-262048. 25342129 PMC4314222

[dneu22984-bib-0045] Kelley, N. , D. Jeltema , Y. Duan , and Y. He . 2019. “The NLRP3 Inflammasome: An Overview of Mechanisms of Activation and Regulation.” International Journal of Molecular Sciences 20, no. 13: 3328. 10.3390/ijms20133328. 31284572 PMC6651423

[dneu22984-bib-0046] Khan, S. , K. H. Barve , and M. S. Kumar . 2020. “Recent Advancements in Pathogenesis, Diagnostics and Treatment of Alzheimer's Disease.” Current Neuropharmacology 18, no. 11: 1106–1125. 10.2174/1570159x18666200528142429. 32484110 PMC7709159

[dneu22984-bib-0047] Kong, L. , D. Zhang , S. Huang , et al. 2023. “Extracellular Vesicles in Mental Disorders: A State‐of‐Art Review.” International Journal of Biological Sciences 19, no. 4: 1094–1109. 10.7150/ijbs.79666. 36923936 PMC10008693

[dneu22984-bib-0048] Kulichikhin, K. Y. , S. A. Fedotov , M. S. Rubel , et al. 2021. “Development of Molecular Tools for Diagnosis of Alzheimer's Disease That Are Based on Detection of Amyloidogenic Proteins.” Prion 15, no. 1: 56–69. 10.1080/19336896.2021.1917289. 33910450 PMC8096329

[dneu22984-bib-0049] Kumar, A. , H. S. Bhatia , A. C. de Oliveira , and B. L. Fiebich . 2015. “microRNA‐26a Modulates Inflammatory Response Induced by Toll‐Like Receptor 4 Stimulation in Microglia.” Journal of Neurochemistry 135, no. 6: 1189–1202. 10.1111/jnc.13364. 26376347

[dneu22984-bib-0050] Lehmann, S. M. , C. Kruger , B. Park , et al. 2012. “An Unconventional Role for miRNA: Let‐7 Activates Toll‐Like Receptor 7 and Causes Neurodegeneration.” Nature Neuroscience 15, no. 6: 827–835. 10.1038/nn.3113. 22610069

[dneu22984-bib-0051] Li, X. , Y. Zhu , Y. Wang , X. Xia , and J. C. Zheng . 2023. “Neural Stem/Progenitor Cell‐Derived Extracellular Vesicles: A Novel Therapy for Neurological Diseases and Beyond.” MedComm (2020) 4, no. 1: e214. 10.1002/mco2.214. 36776763 PMC9905070

[dneu22984-bib-0052] Li, Y. , Y. Gui , M. Zhao , et al. 2023. “The Roles of Extracellular Vesicles in Major Depressive Disorder.” Frontiers in Psychiatry 14: 1138110. 10.3389/fpsyt.2023.1138110. 36970289 PMC10033661

[dneu22984-bib-0053] Li, Y. , S. Meng , W. Di , et al. 2022. “Amyloid‐Beta Protein and MicroRNA‐384 in NCAM‐Labeled Exosomes From Peripheral Blood Are Potential Diagnostic Markers for Alzheimer's Disease.” CNS Neuroscience & Therapeutics 28, no. 7: 1093–1107. 10.1111/cns.13846. 35470961 PMC9160455

[dneu22984-bib-0054] Li, Y. , J. Zhang , J. Wan , A. Liu , and J. Sun . 2020. “Melatonin Regulates Abeta Production/Clearance Balance and Abeta Neurotoxicity: A Potential Therapeutic Molecule for Alzheimer's Disease.” Biomedicine & Pharmacotherapy 132: 110887. 10.1016/j.biopha.2020.110887. 33254429

[dneu22984-bib-0055] Liang, Y. , and L. Wang . 2021. “Inflamma‐MicroRNAs in Alzheimer's Disease: From Disease Pathogenesis to Therapeutic Potentials.” Frontiers in Cellular Neuroscience 15: 785433. 10.3389/fncel.2021.785433. 34776873 PMC8581643

[dneu22984-bib-0056] Lin, J. , J. Li , B. Huang , et al. 2015. “Exosomes: Novel Biomarkers for Clinical Diagnosis.” Thescientificworldjournal [Electronic Resource] 2015: 657086. 10.1155/2015/657086. 25695100 PMC4322857

[dneu22984-bib-0057] Losurdo, M. , and M. Grilli . 2020. “Extracellular Vesicles, Influential Players of Intercellular Communication Within Adult Neurogenic Niches.” International Journal of Molecular Sciences 21, no. 22: 8819. 10.3390/ijms21228819. 33233420 PMC7700666

[dneu22984-bib-0058] Lu, T. , L. Aron , J. Zullo , et al. 2014. “REST and Stress Resistance in Ageing and Alzheimer's Disease.” Nature 507, no. 7493: 448–454. 10.1038/nature13163. 24670762 PMC4110979

[dneu22984-bib-0059] Luo, D. , H. Liu , H. Liu , et al. 2023. “Long RNA Profiles of Human Brain Extracellular Vesicles Provide New Insights Into the Pathogenesis of Alzheimer's Disease.” Aging and Disease 14, no. 1: 229–244. 10.14336/AD.2022.0607. 36818567 PMC9937700

[dneu22984-bib-0060] Ma, R. , L. Chen , N. Hu , S. Caplan , and G. Hu . 2024. “Cilia and Extracellular Vesicles in Brain Development and Disease.” Biological Psychiatry 95, no. 11: 1020–1029. 10.1016/j.biopsych.2023.11.004. 37956781 PMC11087377

[dneu22984-bib-0061] Ma, Y. , C. Li , Y. Huang , Y. Wang , X. Xia , and J. C. Zheng . 2019. “Exosomes Released From Neural Progenitor Cells and Induced Neural Progenitor Cells Regulate Neurogenesis Through miR‐21a.” Cell Communication and Signaling 17, no. 1: 96. 10.1186/s12964-019-0418-3. 31419975 PMC6698014

[dneu22984-bib-0062] Manni, G. , S. Buratta , M. T. Pallotta , et al. 2023. “Extracellular Vesicles in Aging: An Emerging Hallmark?” Cells 12, no. 4: 527. 10.3390/cells12040527. 36831194 PMC9954704

[dneu22984-bib-0063] Marzola, P. , T. Melzer , E. Pavesi , J. Gil‐Mohapel , and P. S. Brocardo . 2023. “Exploring the Role of Neuroplasticity in Development, Aging, and Neurodegeneration.” Brain Sciences 13, no. 12: 1610. 10.3390/brainsci13121610. 38137058 PMC10741468

[dneu22984-bib-0064] Mathivanan, S. , H. Ji , and R. J. Simpson . 2010. “Exosomes: Extracellular Organelles Important in Intercellular Communication.” Journal of Proteomics 73, no. 10: 1907–1920. 10.1016/j.jprot.2010.06.006. 20601276

[dneu22984-bib-0065] Meneses, A. , S. Koga , J. O'Leary , D. W. Dickson , G. Bu , and N. Zhao . 2021. “TDP‐43 Pathology in Alzheimer's Disease.” Molecular Neurodegeneration 16, no. 1: 84. 10.1186/s13024-021-00503-x. 34930382 PMC8691026

[dneu22984-bib-0066] Morton, M. C. , V. N. Neckles , C. M. Seluzicki , J. C. Holmberg , and D. M. Feliciano . 2018. “Neonatal Subventricular Zone Neural Stem Cells Release Extracellular Vesicles That Act as a Microglial Morphogen.” Cell Reports 23, no. 1: 78–89. 10.1016/j.celrep.2018.03.037. 29617675

[dneu22984-bib-0067] Nam, E. , Y. B. Lee , C. Moon , and K. A. Chang . 2020. “Serum Tau Proteins as Potential Biomarkers for the Assessment of Alzheimer's Disease Progression.” International Journal of Molecular Sciences 21, no. 14: 5007. 10.3390/ijms21145007. 32679907 PMC7404390

[dneu22984-bib-0068] Nogueras‐Ortiz, C. J. , V. Mahairaki , F. Delgado‐Peraza , et al. 2020. “Astrocyte‐ and Neuron‐Derived Extracellular Vesicles From Alzheimer's Disease Patients Effect Complement‐Mediated Neurotoxicity.” Cells 9, no. 7: 1618. 10.3390/cells9071618. 32635578 PMC7407141

[dneu22984-bib-0069] Park, S. Y. , D. S. Kim , H. M. Kim , et al. 2022. “Human Mesenchymal Stem Cell‐Derived Extracellular Vesicles Promote Neural Differentiation of Neural Progenitor Cells.” International Journal of Molecular Sciences 23, no. 13: 7047. 10.3390/ijms23137047. 35806058 PMC9267053

[dneu22984-bib-0070] Parkash, S. , and K. Radhakrishna . 1987. “Ventrally Based and Turned in Dermofat Pedicled Flaps in Repair of Abdominal Wall and Thoracic Defects.” British Journal of Plastic Surgery 40, no. 1: 31–36. 10.1016/0007-1226(87)90007-5. 2949792

[dneu22984-bib-0071] Perea, J. R. , J. Avila , and M. Bolos . 2018. “Dephosphorylated Rather Than Hyperphosphorylated Tau Triggers a Pro‐Inflammatory Profile in Microglia Through the p38 MAPK Pathway.” Experimental Neurology 310: 14–21. 10.1016/j.expneurol.2018.08.007. 30138606

[dneu22984-bib-0072] Pounders, J. , E. J. Hill , D. Hooper , et al. 2022. “MicroRNA Expression Within Neuronal‐Derived Small Extracellular Vesicles in Frontotemporal Degeneration.” Medicine 101, no. 40: e30854. 10.1097/MD.0000000000030854. 36221381 PMC9542922

[dneu22984-bib-0114] Rodriguez‐Grande, B. , L. Varghese , F. Molina‐Holgado , et al. 2015. “Pentraxin 3 mediates neurogenesis and angiogenesis after cerebral ischaemia.” J Neuroinflammation, 12: 15. https://doi.10.1186/s12974‐014‐0227‐y. 25616391 10.1186/s12974-014-0227-y PMC4308938

[dneu22984-bib-0073] Ruan, Z. 2022. “Extracellular Vesicles Drive Tau Spreading in Alzheimer's Disease.” Neural Regeneration Research 17, no. 2: 328–329. 10.4103/1673-5374.317975. 34269203 PMC8463965

[dneu22984-bib-0113] Ruan, Z. , J. C. Delpech , S. Venkatesan Kalavai , et al. 2020. “P2RX7 inhibitor suppresses exosome secretion and disease phenotype in P301S tau transgenic mice.” Mol Neurodegener, 15, no. 1: 47. https://doi.10.1186/s13024‐020‐00396‐2. 32811520 10.1186/s13024-020-00396-2 PMC7436984

[dneu22984-bib-0074] Rufino‐Ramos, D. , S. Lule , S. Mahjoum , et al. 2022. “Using Genetically Modified Extracellular Vesicles as a Non‐Invasive Strategy to Evaluate Brain‐Specific Cargo.” Biomaterials 281: 121366. 10.1016/j.biomaterials.2022.121366. 35033904 PMC8886823

[dneu22984-bib-0075] Sardar Sinha, M. , A. Ansell‐Schultz , L. Civitelli , et al. 2018. “Alzheimer's Disease Pathology Propagation by Exosomes Containing Toxic Amyloid‐Beta Oligomers.” Acta Neuropathologica 136, no. 1: 41–56. 10.1007/s00401-018-1868-1. 29934873 PMC6015111

[dneu22984-bib-0076] Schiera, G. , P. Proia , C. Alberti , M. Mineo , G. Savettieri , and I. Di Liegro . 2007. “Neurons Produce FGF2 and VEGF and Secrete Them at Least in Part by Shedding Extracellular Vesicles.” Journal of Cellular and Molecular Medicine 11, no. 6: 1384–1394. 10.1111/j.1582-4934.2007.00100.x. 18205708 PMC4401300

[dneu22984-bib-0077] Serpente, M. , C. Fenoglio , M. D'Anca , et al. 2020. “MiRNA Profiling in Plasma Neural‐Derived Small Extracellular Vesicles From Patients With Alzheimer's Disease.” Cells 9, no. 6: 1443. 10.3390/cells9061443. 32531989 PMC7349735

[dneu22984-bib-0078] Sharma, P. , L. Schiapparelli , and H. T. Cline . 2013. “Exosomes Function in Cell‐Cell Communication During Brain Circuit Development.” Current Opinion in Neurobiology 23, no. 6: 997–1004. 10.1016/j.conb.2013.08.005. 23998929 PMC3830597

[dneu22984-bib-0079] Shi, M. , C. Liu , T. J. Cook , et al. 2014. “Plasma Exosomal Alpha‐Synuclein Is Likely CNS‐Derived and Increased in Parkinson's Disease.” Acta Neuropathologica 128, no. 5: 639–650. 10.1007/s00401-014-1314-y. 24997849 PMC4201967

[dneu22984-bib-0080] Shin, T. S. , J. H. Kim , Y. S. Kim , et al. 2010. “Extracellular Vesicles Are Key Intercellular Mediators in the Development of Immune Dysfunction to Allergens in the Airways.” Allergy 65, no. 10: 1256–1265. 10.1111/j.1398-9995.2010.02359.x. 20337607 PMC3066408

[dneu22984-bib-0081] Singh, R. , S. Rai , P. S. Bharti , et al. 2024. “Circulating Small Extracellular Vesicles in Alzheimer's Disease: A Case‐Control Study of Neuro‐Inflammation and Synaptic Dysfunction.” BMC Medicine [Electronic Resource] 22, no. 1: 254. 10.1186/s12916-024-03475-z. 38902659 PMC11188177

[dneu22984-bib-0082] Song, Z. , Y. Xu , W. Deng , et al. 2020. “Brain Derived Exosomes Are a Double‐Edged Sword in Alzheimer's Disease.” Frontiers in Molecular Neuroscience 13: 79. 10.3389/fnmol.2020.00079. 32547364 PMC7274346

[dneu22984-bib-0083] Stevanato, L. , L. Thanabalasundaram , N. Vysokov , and J. D. Sinden . 2016. “Investigation of Content, Stoichiometry and Transfer of miRNA From Human Neural Stem Cell Line Derived Exosomes.” PLoS ONE 11, no. 1: e0146353. 10.1371/journal.pone.0146353. 26752061 PMC4713432

[dneu22984-bib-0084] Stronati, E. , R. Conti , E. Cacci , S. Cardarelli , S. Biagioni , and G. Poiana . 2019. “Extracellular Vesicle‐Induced Differentiation of Neural Stem Progenitor Cells.” International Journal of Molecular Sciences 20, no. 15: 3691. 10.3390/ijms20153691. 31357666 PMC6696602

[dneu22984-bib-0085] Sullivan, L. B. , D. Y. Gui , A. M. Hosios , L. N. Bush , E. Freinkman , and M. G. Vander Heiden . 2015. “Supporting Aspartate Biosynthesis Is an Essential Function of Respiration in Proliferating Cells.” Cell 162, no. 3: 552–563. 10.1016/j.cell.2015.07.017. 26232225 PMC4522278

[dneu22984-bib-0086] Szepesi, Z. , O. Manouchehrian , S. Bachiller , and T. Deierborg . 2018. “Bidirectional Microglia‐Neuron Communication in Health and Disease.” Frontiers in Cellular Neuroscience 12: 323. 10.3389/fncel.2018.00323. 30319362 PMC6170615

[dneu22984-bib-0087] Tasdelen, E. , E. T. Ozel Kizil , S. Tezcan , E. Yalap , A. P. Bingol , and N. Y. Kutlay . 2022. “Determination of miR‐373 and miR‐204 Levels in Neuronal Exosomes in Alzheimer's Disease.” Turkish Journal of Medical Sciences 52, no. 5: 1458–1467. 10.55730/1300-0144.5484. 36422510 PMC10395704

[dneu22984-bib-0088] Vingtdeux, V. , M. Hamdane , A. Loyens , et al. 2007. “Alkalizing Drugs Induce Accumulation of Amyloid Precursor Protein By‐Products in Luminal Vesicles of Multivesicular Bodies.” Journal of Biological Chemistry 282, no. 25: 18197–18205. 10.1074/jbc.M609475200. 17468104

[dneu22984-bib-0089] Wallin, A. K. , K. Blennow , N. Andreasen , and L. Minthon . 2006. “CSF Biomarkers for Alzheimer's Disease: Levels of Beta‐Amyloid, Tau, Phosphorylated Tau Relate to Clinical Symptoms and Survival.” Dementia and Geriatric Cognitive Disorders 21, no. 3: 131–138. 10.1159/000090631. 16391474

[dneu22984-bib-0090] Wang, X. , H. Yang , C. Liu , and K. Liu . 2023. “A New Diagnostic Tool for Brain Disorders: Extracellular Vesicles Derived From Neuron, Astrocyte, and Oligodendrocyte.” Frontiers in Molecular Neuroscience 16: 1194210. 10.3389/fnmol.2023.1194210. 37621405 PMC10445044

[dneu22984-bib-0091] Wang, Y. , V. Balaji , S. Kaniyappan , et al. 2017. “The Release and Trans‐Synaptic Transmission of Tau Via Exosomes.” Molecular Neurodegeneration 12, no. 1: 5. 10.1186/s13024-016-0143-y. 28086931 PMC5237256

[dneu22984-bib-0092] Winston, C. N. , E. J. Goetzl , J. C. Akers , et al. 2016. “Prediction of Conversion From Mild Cognitive Impairment to Dementia With Neuronally Derived Blood Exosome Protein Profile.” Alzheimer's & Dementia (AMST) 3: 63–72. 10.1016/j.dadm.2016.04.001. PMC4925777 27408937

[dneu22984-bib-0093] Winston, C. N. , E. J. Goetzl , L. D. Baker , M. V. Vitiello , and R. A. Rissman . 2018. “Growth Hormone‐Releasing Hormone Modulation of Neuronal Exosome Biomarkers in Mild Cognitive Impairment.” Journal of Alzheimer's Disease 66, no. 3: 971–981. 10.3233/JAD-180302. PMC6487872 30372675

[dneu22984-bib-0094] Xie, Y. , S. He , and J. Wang . 2018. “MicroRNA‐373 Facilitates HSV‐1 Replication Through Suppression of Type I IFN Response by Targeting IRF1.” Biomedicine & Pharmacotherapy 97: 1409–1416. 10.1016/j.biopha.2017.11.071. 29156530

[dneu22984-bib-0095] Xu, G. , J. Chen , G. Jing , and A. Shalev . 2013. “Thioredoxin‐Interacting Protein Regulates Insulin Transcription Through microRNA‐204.” Nature Medicine 19, no. 9: 1141–1146. 10.1038/nm.3287. PMC3835787 23975026

[dneu22984-bib-0096] Yanamandra, K. , N. Kfoury , H. Jiang , et al. 2013. “Anti‐tau Antibodies That Block Tau Aggregate Seeding In Vitro Markedly Decrease Pathology and Improve Cognition In Vivo.” Neuron 80, no. 2: 402–414. 10.1016/j.neuron.2013.07.046. 24075978 PMC3924573

[dneu22984-bib-0097] Yang, L. , F. Niu , H. Yao , et al. 2018. “Exosomal miR‐9 Released From HIV Tat Stimulated Astrocytes Mediates Microglial Migration.” Journal of Neuroimmune Pharmacology 13, no. 3: 330–344. 10.1007/s11481-018-9779-4. 29497921 PMC6082702

[dneu22984-bib-0098] Yao, H. , R. Ma , L. Yang , et al. 2014. “MiR‐9 Promotes Microglial Activation by Targeting MCPIP1.” Nature Communications 5: 4386. 10.1038/ncomms5386. PMC4104446 25019481

[dneu22984-bib-0099] Yerrapragada, S. M. , and J. C. Bihl . 2022. “Role of Exosomes in Mediating the Cross‐Talk Between Adipose Tissue and the Brain.” Neuromolecular Medicine 24, no. 2: 57–61. 10.1007/s12017-021-08664-0. 33978939 PMC8674931

[dneu22984-bib-0100] Yin, Z. , Z. Han , T. Hu , et al. 2020. “Neuron‐Derived Exosomes With High miR‐21‐5p Expression Promoted Polarization of M1 Microglia in Culture.” Brain, Behavior, and Immunity 83: 270–282. 10.1016/j.bbi.2019.11.004. 31707083

[dneu22984-bib-0101] Yuan, P. , L. Ding , H. Chen , et al. 2021. “Neural Stem Cell‐Derived Exosomes Regulate Neural Stem Cell Differentiation Through miR‐9‐Hes1 Axis.” Frontiers in Cell and Developmental Biology 9: 601600. 10.3389/fcell.2021.601600. 34055767 PMC8155619

[dneu22984-bib-0102] Yuyama, K. , and Y. Igarashi . 2017. “Exosomes as Carriers of Alzheimer's Amyloid‐ss.” Frontiers in Neuroscience 11: 229. 10.3389/fnins.2017.00229. 28487629 PMC5403946

[dneu22984-bib-0103] Yuyama, K. , H. Sun , S. Usuki , et al. 2015. “A Potential Function for Neuronal Exosomes: Sequestering Intracerebral Amyloid‐Beta Peptide.” FEBS Letters 589, no. 1: 84–88. 10.1016/j.febslet.2014.11.027. 25436414

[dneu22984-bib-0104] Zhang, G. , J. Li , S. Purkayastha , et al. 2013. “Hypothalamic Programming of Systemic Ageing Involving IKK‐beta, NF‐kappaB and GnRH.” Nature 497, no. 7448: 211–216. 10.1038/nature12143. 23636330 PMC3756938

[dneu22984-bib-0105] Zhang, H. , Y. Cao , L. Ma , Y. Wei , and H. Li . 2021. “Possible Mechanisms of Tau Spread and Toxicity in Alzheimer's Disease.” Frontiers in Cell and Developmental Biology 9: 707268. 10.3389/fcell.2021.707268. 34395435 PMC8355602

[dneu22984-bib-0106] Zhang, J. , P. Rong , L. Zhang , et al. 2021. “IL4‐Driven Microglia Modulate Stress Resilience Through BDNF‐Dependent Neurogenesis.” Science Advances 7, no. 12: eabb9888. 10.1126/sciadv.abb9888. 33731342 PMC7968840

[dneu22984-bib-0107] Zhang, L. , Y. J. Li , X. Y. Wu , Z. Hong , and W. S. Wei . 2015. “MicroRNA‐181c Negatively Regulates the Inflammatory Response in Oxygen‐Glucose‐Deprived Microglia by Targeting Toll‐Like Receptor 4.” Journal of Neurochemistry 132, no. 6: 713–723. 10.1111/jnc.13021. 25545945

[dneu22984-bib-0108] Zhang, N. , D. Gu , M. Meng , and M. L. Gordon . 2020. “TDP‐43 Is Elevated in Plasma Neuronal‐Derived Exosomes of Patients with Alzheimer's Disease.” Frontiers in Aging Neuroscience 12: 166. 10.3389/fnagi.2020.00166. 32581773 PMC7287025

[dneu22984-bib-0109] Zhang, W. , B. Zhong , C. Zhang , C. Luo , and Y. Zhan . 2018. “miR‐373 Regulates Inflammatory Cytokine‐Mediated Chondrocyte Proliferation in Osteoarthritis by Targeting the P2×7 Receptor.” FEBS Open Bio 8, no. 3: 325–331. 10.1002/2211-5463.12345. PMC5832977 29511609

[dneu22984-bib-0110] Zhang, Y. , M. S. Kim , B. Jia , et al. 2017. “Hypothalamic Stem Cells Control Ageing Speed Partly Through Exosomal miRNAs.” Nature 548, no. 7665: 52–57. 10.1038/nature23282. 28746310 PMC5999038

[dneu22984-bib-0111] Zhao, A. , Y. Li , Y. Yan , et al. 2020. “Increased Prediction Value of Biomarker Combinations for the Conversion of Mild Cognitive Impairment to Alzheimer's Dementia.” Translational Neurodegeneration 9, no. 1: 30. 10.1186/s40035-020-00210-5. 32741361 PMC7397685

[dneu22984-bib-0112] Zhong, J. , X. Ren , W. Liu , et al. 2021. “Discovery of Novel Markers for Identifying Cognitive Decline Using Neuron‐Derived Exosomes.” Frontiers in Aging Neuroscience 13: 696944. 10.3389/fnagi.2021.696944. 34512304 PMC8427802

